# A Semi-Analytical Solution for Shock Wave Pressure and Radius of Soil Plastic Zone Induced by Lightning Strikes

**DOI:** 10.3390/ma15062239

**Published:** 2022-03-17

**Authors:** Zhilin Wu, Pingping Rao, Sanjay Nimbalkar, Qingsheng Chen, Jifei Cui, Peihao Ouyang

**Affiliations:** 1 Department of Civil Engineering, University of Shanghai for Science and Technology, 516 Jungong Road, Shanghai 200093, China; 192581774@st.usst.edu.cn (Z.W.); cuijifei@usst.edu.cn (J.C.); 203642074@st.usst.edu.cn (P.O.); 2 School of Civil and Environmental Engineering, University of Technology Sydney, 15 Broadway, Ultimo, NSW 2007, Australia; sanjay.nimbalkar@uts.edu.au; 3 Technology Research Center of Ecological Road Engineering, Hubei University of Technology, Wuhan 430068, China; chqsh2006@163.com

**Keywords:** semi-analytical solution, lightning strikes, shock wave pressure, radius of soil plastic zone, plasma explosive

## Abstract

A semi-analytical solution for forecasting the soil behavior induced by lightning strikes is of great engineering significance to calculate the radius of the soil plastic zone. In this paper, a simplified two-stage method is employed to solve the shock wave pressure and the radius of the soil plastic zone. The solution is verified against experimental data. Using the present model, the major factors dominating the shock wave pressure and the radius of the soil plastic zone are investigated. The results show that (1) the radius of the soil plastic zone (*r_p_*) induced by lightning decreases monotonically with cohesion (*c*) and internal friction angle (*φ*), while *c* has a better effect on soil properties than *φ* does; (2) increasing the initial radius of the plasma channel (*r_i_*_0_) can reduce the pressure (*P*) and increasing *r_i_*_0_ has a nonnegligible effect on *r_p_*; with *r_i_*_0_ increasing by 100%, the radius of the soil plastic zone increases by 47.9–59.7%; (3) the plasma channel length (*L*) has a significant influence on *P* and *r_p_*, especially when *L* is at a relatively low level; (4) the *r_p_* induced by lightning decreases exponentially with attenuation coefficient (*a*); (5) the wavefront time is a major factor while the half-value time is a minor factor for the shock wave pressure induced by plasma explosives.

## 1. Introduction

**Figure 1:**
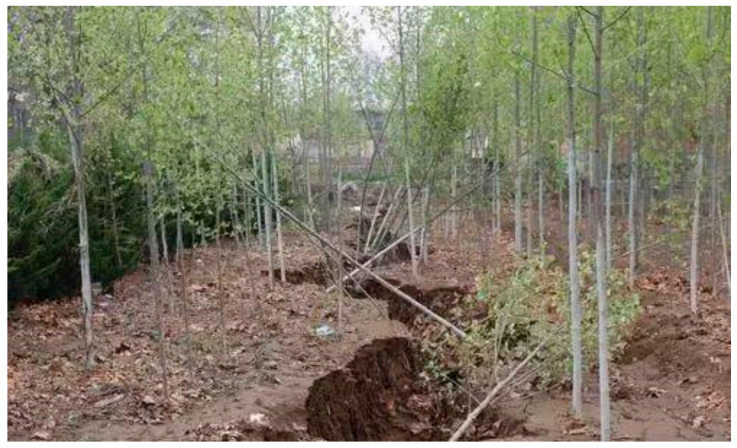
Cracked ground due to lightning strikes in Yuncheng, Shanxi, China.

**Figure 2:**
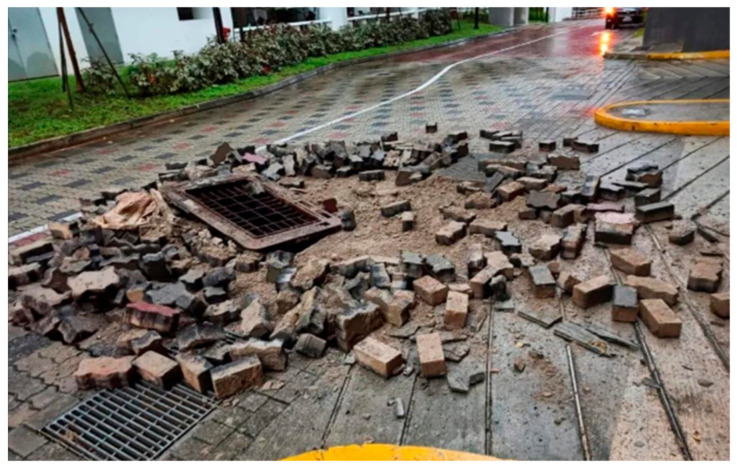
Pothole due to lightning strikes in Batok Town, Bukit, Singapore.

Lightning is a dangerous, frequent geophysical phenomenon [1,2], and large amounts of heat and electricity are produced by considerable energy and intense electromagnetic field activities [3,4]. The lightning forms a plasma channel that propagates outward from its starting point with positive and negative charges at each end, known as positive and negative leaders, respectively [3]. For example, lightning caused a 50 m long and 1 m wide breach in the ground in Yuncheng, Shanxi Province, China and caused a pothole in Batok Town, Bukit, Singapore (see Figure 1 and Figure 2, respectively) [5,6]. Lightning struck buried pipelines in Alabama and Cideville, France, both causing pipeline leaks and posing a significant threat to pipeline transportation [7,8]. Lightning struck an airport runway on the west coast of India, causing damage to the runway as well as cracks to the roadbed [9]. In the event of a lightning strike, the soil serves as a significant dissipation medium and its dispersion qualities are critical [10,11,12]. Thus, it is necessary to investigate the principle of soil plastic deformation induced by lightning strikes.

Thus, it is necessary to solve the shock wave pressure induced by lightning. Meanwhile, lightning is a special form of electrical impulse. Some studies in the literature provided analytical solutions to solve the process of plasma explosives. Drabkina et al. [13] carried out innovative work in the arc channel expansion of pulsed gas discharges; however, their model cannot calculate the conductivity and temperature change of the arc channel. Braginskii et al. [14] proposed an arc channel energy equation to study the arc channel expansion, but they assumed that the current is proportional to time, so the calculation can only be used in the arc channel expansion initial stage. Based on the work of Drabkina et al. [13] and Braginskii et al. [14], Engel et al. [15] proposed a method for calculating the channel radius with the arc current as the independent variable, and gave a pressure–time relationship. From the arc channel collision expression of Engel et al. [15], which shows that the arc channel radius increases with time, it is not in line with the actual development of the arc channel law. Numerous analytical solutions have been provided to forecast water shock waves induced by arc discharges [16,17,18,19,20,21]. A physical and mathematical model of electro bursts has been provided to forecast the shock wave pressure generated by the expanding plasma channel [22,23]. Burkin et al. [24] proposed a model for electric pulse rock breaking; however, the model assumes that the plasma channel is a cylinder of constant length, which needs to be improved in subsequent research.

The studies mentioned above have provided analytical solutions of the shock wave pressure induced by arc discharges in air, water, and solid media. However, the equation for the pressure in soil has not been given. A physical and mathematical model of rock breaking has been provided to study the mechanism of high-voltage pulsed rock fragmentation [25,26,27]. Li et al. [28] investigated the electro-dynamic mechanisms of electric breakdown by using the time-transient breakdown model and the finite-difference numerical method. Peng et al. [29] used experiments and numerical simulations to investigate the causes of damage to rocks caused by arc blasts and concluded that the surrounding pressure is the main factor affecting rock cracking, but they did not give a quantitative evaluation index. Pulse discharge technology is an innovative technique that has been used to enhance the bearing capacity of piles, resisting capacity of anchors, and rock-breaking drilling [30,31,32]. Walsh et al. [33] simulated the passage of the pulse, electrical breakdown in the rock, and the mechanical response at the grain-scale by building a multiphysics model, but this model does not implement the mutual coupling between the electric-thermal force. Chen et al. [34] investigated the thermal and impact effects of lightning on rock, using Joule heat theory to obtain the surface temperature distribution of granite, but they did not consider the effect of the current waveform on shockwave pressure. Yan et al. [35] investigated the breakdown process and fragmentation characteristics of anthracite through high-voltage electrical pulse treatment, but they did not explain the phenomenon well.

The purpose of this paper is to provide a semi-analytical solution based on the work of Rao et al. [36,37] for solving the shock wave pressure and the radius of the soil plastic zone (RSPZ) caused by lightning strikes. The proposed solution is verified against the experimental data. Considering the equation of state of the soil, the Runge–Kutta method is applied to solve the governing equation for shock wave pressure. Then, the modified Mohr–Coulomb yield criterion is applied to solve the RSPZ. The major factors dominating the shock wave pressure and the RSPZ are investigated. Soil is a kind of mineral material, and this paper can provide a further understanding of the mechanical properties of soil after lightning strikes and can provide some guidance for lightning mitigation.

## 2. Mathematical Model and Analytical Solution

### 2.1. Geometric Model and Simplifying Assumption

**Figure 3:**
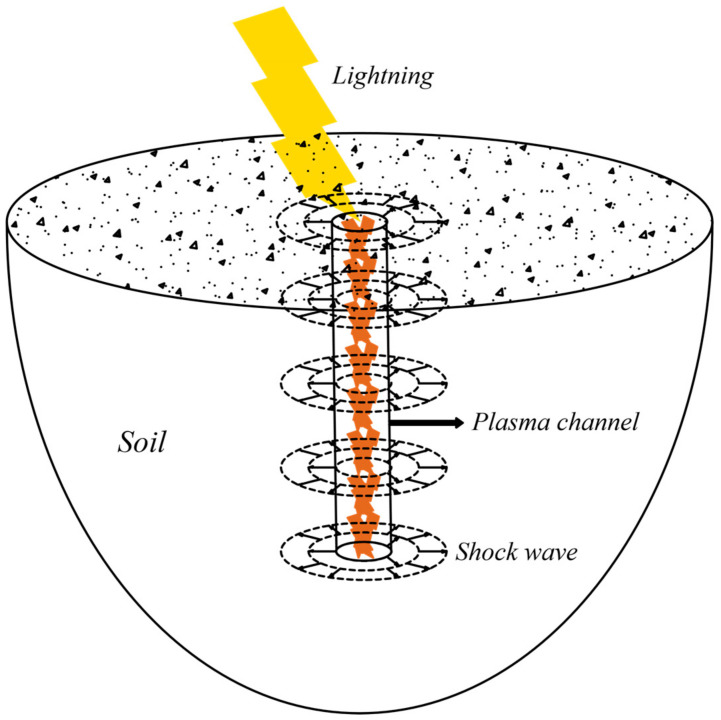
Mathematical model for soil deformation through lightning strikes.

Figure 3 shows the geometric deformation model of soil by lightning impact. The system is composed of four parts, i.e., lightning, plasma channel, shock wave, and soil. The principle of the plasma channel in soil is introduced by the theory of electrical breakdown of solid dielectrics. When the plasma channel is formed, the energy in the pulse power source is released into the plasma channel (up to 10^4^ K) and the channel is heated. Then, the plasma channel is rapidly expanded, generates shock waves to act on the surrounding soil, and finally leads to the deformation of soil [25].

Several suppositions are applied to assist in the solving of the analytical solution: (1) a standard double index current of lightning is assumed to be fixed at expression *I*(*t*) [38]; (2) the property of soil is assumed to isotropic; (3) the path of the plasma channel is assumed to be a line, i.e., not including branches of a channel.

### 2.2. Governing Equations

#### 2.2.1. Lightning Energy Diffusion Model

**Figure 4:**
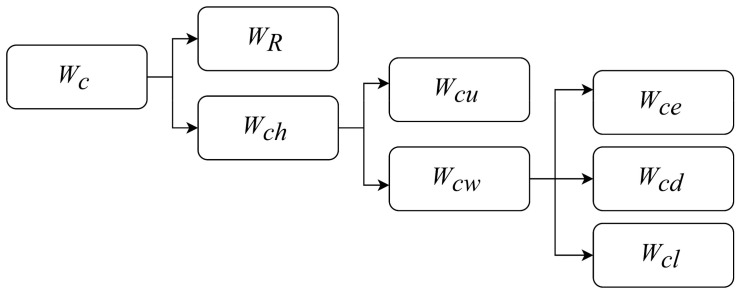
Schematics of energy conversion.

When a lightning pulse discharge occurs, the energy in the pulse power source is released into the plasma channel and converted into other forms energy. The schematic of energy conversion is shown in Figure 4. The total energy of (*W_c_*) is mainly consumed by resistance loss in the electrical circuit (*W_R_*) and the energy injected into the plasma channel *W_ch_*. The energy injected into the plasma channel (*W_ch_*) is converted to the plasma channel heat energy (*W_cu_*) and the shock wave energy (*W_cw_*) to work with channel expansion, wherein the shockwave energy *W_cw_* consists of internal energy (*W_ce_*), kinetic energy (*W_cd_*), and reflected wave energy (*W_cl_*) [22].

The channel energy balance equation for *W_ch_*, *W_cu_,* and *W_cw_* is a key equation to connect plasma channel energy and the discharge energy converted into plasma channel heat energy and mechanical work. The relationship between *W_ch_*, *W_cu_,* and *W_cw_* has been described by Burkin et al. [22].

(1) $$\frac{dW_{ch}}{dt}=\frac{dW_{cw}}{dt}+\frac{d(P_{ch}V_{ch})}{(\gamma -1)dt}$$

where *dW_cw_* = *P_ch_***·***dV_ch_* describes the channel work by the expanding channel at its volume *V_ch_* = π*r*^2^*_ch_*(*t*)*l_ch_* with pressure *P_ch_*, where *r_ch_*(*t*) is the channel radius. *W_cu_* = *P_ch_V_ch_*(*γ* − 1)^−1^ describes the plasma channel heat energy, where *γ* is the effective ratio of specific heats.

The governing equation of the energy injected into the plasma channel *W_ch_* is [23]

(2) $$W_{ch}=\int_{0}^{t}i^{2}\left(\tau \right)R_{ch}(\tau )d\tau$$

where *i*(*t*) is the lightning pulse current and *R_ch_*(*t*) is the breakdown channel resistance.

The breakdown channel resistance adopts the Weizel–Rompe model of impedance [39]. The equation of impedance of the Weizel–Rompe model is [22,23,39].

(3) $$R_{ch}(t){=K}_{ch}\cdot l_{ch}{\left(\int_{0}^{t}i^{2}(\tau )d\tau \right)}^{-\frac{1}{2}}$$

where *K_ch_* is a spark constant and *l_ch_* is the length of the plasma channel.

The double exponential current expressions *i*(*t*) is

(4) $${i(t)=i}_{m}k_{m}(e^{-\alpha t}{-e}^{-\beta t})$$

where *i_m_* is the peak current, *k_m_* is the correction coefficient, *α* is the wavefront coefficient, and *β* is the half-value coefficient of the double exponential current.

Substituting the double exponential current expressions *i*(*t*) into Equation (3):

(5) $$R_{ch}(t){=K}_{ch}\cdot l_{ch}{\left(\int_{0}^{t}\left(i_{m}k_{m}{(e}^{-\alpha \tau }{-e}^{-\beta \tau }\right)\right)}^{2}d\tau {)}^{-\frac{1}{2}}$$

When solving the problems in the circuits comprising only the spark channel as a load, Equation (5) gives *R_ch_* = *∞* at *t* = 0, which makes the initial conditions uncertain. In this paper, the approximation is

(6) $$R_{ch}{(t)=K}_{ch}\cdot l_{ch}{(\Delta +\int_{0}^{t}{(i}_{m}k_{m}{(e}^{-\alpha \tau }{-e}^{-\beta \tau }))}^{2}{d\tau )}^{-\frac{1}{2}}$$

where ∆ ≈ π*K_ch_r*_0_*σ*_0_, *r*_0_ is the initial radius of the plasma channel, and *σ*_0_ is the electrical conductivity averaged over the cross-section of the plasma channel.

#### 2.2.2. Stress of Shock Wave Model

**Figure 5:**
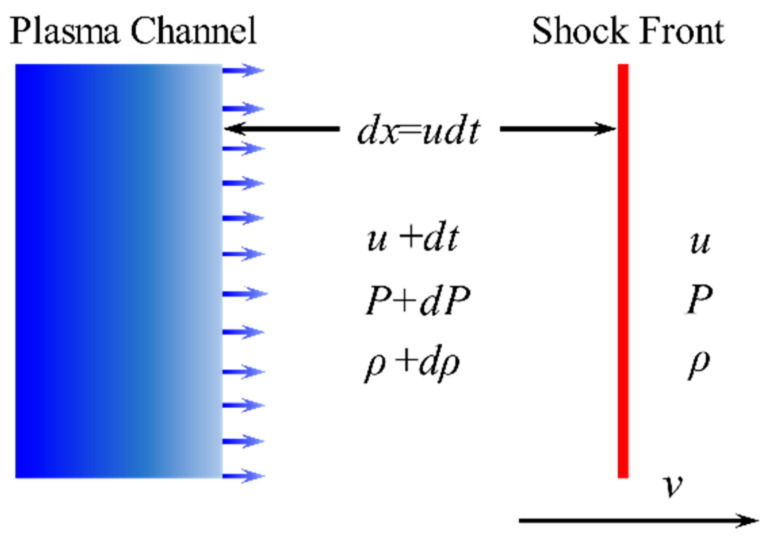
Plasma channel expansion.

A relationship between the pressure on the surface of the channel and the velocity of the channel wall is derived from the Rankine–Hugoniot relations for a shock front [40]. For soil solids, the plasma channel generated by a lightning pulse in the solid can be regarded as an expanding cylindrical piston. Now, consider a piston from which a continuous succession of shock fronts originate as its velocity varies in a continuous manner [16], as shown in Figure 5. The conservation of mass and conservation of momentum at the front is as follows:

(7) $$\rho_{0}{(v-u}_{0}{)=(\rho }_{0}{+d\rho )[v-(u}_{0}+\mathrm{du})]$$

(8) $${dP=\rho (v-u}_{0})du$$

where *ρ* is the density, *u* is the velocity of soil, *v* is the velocity of the shockwave front, *dρ* is the change in density, *du* is the change in velocity, *P* is the pressure, and *dP* is the change in pressure.

To solve the problem of the stress wave, substituting the Equation (7) into Equation (8) and omitting a low-order factor *ρdu*:

(9) $$\frac{dP}{{\left(du\right)}^{2}}=\frac{\rho^{2}}{d\rho }$$

The state equation of Murnaghan is deduced from the definition of bulk modulus [41]. Expanding *K*(*P*) by the Maclaurin series, and taking the first two terms of the expansion, the state equation is as follows:

(10) $$K(P){=K}_{0}{+K}_{0}^{}{}^{*}P$$

(11) $$K(P){=-V}_{ch}\frac{dP}{{dV}_{ch}}$$

where *K*_0_ and *K*_0_^*^ are determined via experiment, and *K*_0_ is the bulk modulus of soil without pressure.

Substituting Equation (10) into Equation (11) followed by integration:

(12) $$P=\psi [{\left(\frac{\rho }{\rho_{0}}\right)}^{n}-1]$$

(13) $$\psi =\frac{K_{0}}{K_{0}^{*}},{\;n=K}_{0}^{*}$$

where *Ψ* and *n* are determined via experiment, *K*_0_ = *ρ*_0_*v*_0_^2^, *n* = 4*s* − 1, *v*_0_ is the material speed of sound, *ρ*_0_ is the density in the undisturbed state, and *s* is the material constant [42].

The governing equation for the problem stress wave would be obtained by substituting Equation (9) into Equation (12) as

(14) $${\left(P+\psi \right)}^{\frac{n-1}{2n}}=\frac{u(n-1)}{2n}\psi^{\frac{-1}{2n}}{\left({n\rho }_{0}\right)}^{\frac{1}{2}}+\psi^{\frac{n-1}{2n}}$$

For the solid materials, substituting *s* = 1.5 into Equation (14) [42]:

(15) $${(P+\psi )}^{\frac{2}{5}}=\frac{2}{\sqrt{5}}\rho_{0}{}^{\frac{1}{2}}\psi^{-\frac{1}{10}}u+\psi^{\frac{2}{5}}$$

According to a simple mathematical transformation:

(16) $$u=\frac{\sqrt{5}}{2}((P+\psi {)}^{\frac{2}{5}}-\psi^{\frac{2}{5}}{)\rho }_{0}^{-\frac{1}{2}}\alpha^{\frac{1}{10}}$$

The plasma channel expansion rate is:

(17) $$u=\frac{dr}{dt}$$

According to the geometric relationship of a cylinder:

(18) $$V_{ch}{=\pi r}_{ch}{\left(t\right)}^{2}l_{ch}$$

The volume of the plasma channel is differentiated against time:

(19) $$\frac{{dV}_{ch}}{dt}=2{\pi r}_{ch}l_{ch}\frac{dr}{dt}$$

The expression of Equation (18) is transformed as follows:

(20) $$r_{ch}(t)={\left[\frac{V_{ch}}{{\pi l}_{ch}}\right]}^{\frac{1}{2}}$$

Substitute Equation (20) into (19) as follows:

(21) $$\frac{{dV}_{ch}}{dt}=2{\left({\pi l}_{ch}V_{ch}\right)}^{\frac{1}{2}}\frac{dt}{dt}$$

The shock wave pressure with the plasma channel volume would be obtained by substituting Equation (16) into Equation (21) as

(22) $$\frac{{dV}_{ch}}{dt}=(5{\pi l}_{ch}{V)}^{\frac{1}{2}}\rho_{0}^{\frac{-1}{2}}\psi^{\frac{1}{10}}[{(P+\psi )}^{\frac{2}{5}}-\psi^{\frac{2}{5}}]$$

Equations (1), (2) and (22) can be solved numerically using the Runge–Kutta methods [43]. Then, the values of the shock wave pressure *P* and the plasma channel radius *r_ch_*(*t*), which vary with time, can be obtained.

#### 2.2.3. Soil Deformation Model

The cylindrical shockwave comes from the lightning pulse. In a solid medium, stress from the lightning pulse is strongly correlated with stress from the blast load [34].

**Figure 6:**
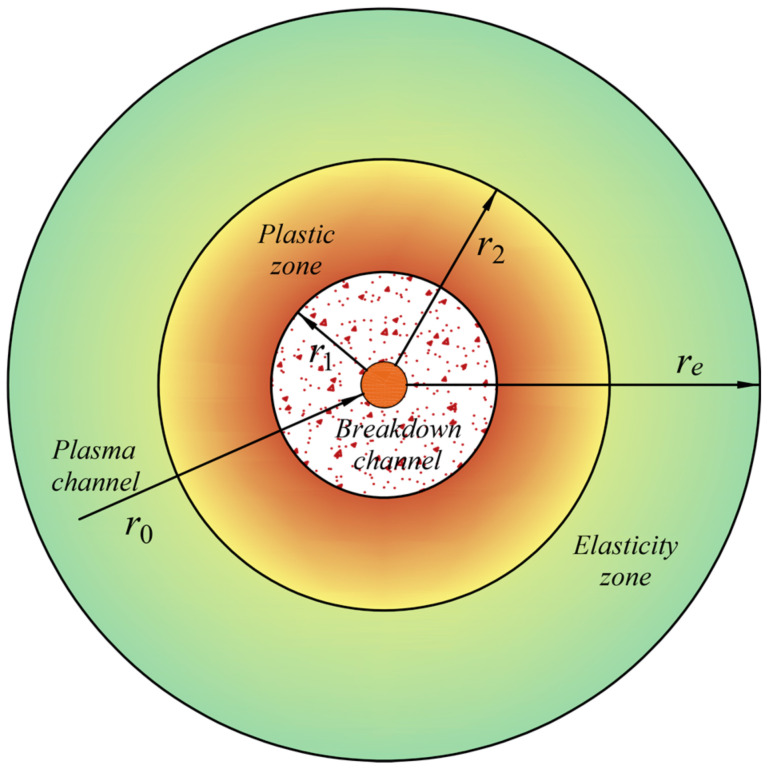
Plasma channel explosion regions.

Figure 6 shows the deformation of soil by shockwave, which divides the soil into three parts: the breakdown channel, plastic zone, and elasticity zone.

Soil is an elastomeric material, and the deformation of soil in the elasticity zone is recoverable as the shockwave gradually decays. The main compaction zone of soil is the plastic zone. According to the theory of soil plasticity mechanics, soil stress in the elasticity zone is [44]

(23) $$\sigma_{r}=\frac{\frac{r_{e}{}^{2}}{r^{2}}-1}{\frac{r_{e}{}^{2}}{r_{2}^{2}}-1}\sigma_{r2}+\frac{1-\frac{r_{2}{}^{2}}{r^{2}}}{1-\frac{r_{2}{}^{2}}{r_{e}^{2}}}\sigma_{re}$$

(24) $$\sigma_{\theta }=-\frac{\frac{r_{e}{}^{2}}{r^{2}}+1}{\frac{r_{e}{}^{2}}{r_{2}^{2}}-1}\sigma_{r2}+\frac{1+\frac{r_{2}{}^{2}}{r^{2}}}{1-\frac{r_{2}{}^{2}}{r_{e}^{2}}}\sigma_{re}$$

When *r_e_*→∞,

(25) $$\sigma_{r}=\frac{r_{2}^{2}}{r^{2}}\sigma_{r2}+(1-\frac{r_{2}{}^{2}}{r^{2}}{)\sigma }_{re}$$

(26) $$\sigma_{\theta }=-\frac{r_{2}^{2}}{r^{2}}\sigma_{r2}+(1+\frac{r_{2}{}^{2}}{r^{2}}{)\sigma }_{re}$$

where *σ_r_* is the radial stress, *r* is the soil radius, *σ_r_*_2_ is the radial stress when *r* = *r*_2_, *σ_θ_* is the cyclic stress, *r_e_* is the radius of a semi-infinite body, as shown in Figure 6, and *σ_r_*_2_ is the radial stress when *r* = *r_e_*.

The plastic zone satisfies the Mohr–Coulomb yield criterion:

(27) $$\sigma_{r}{-\sigma }_{\theta }{=(\sigma }_{r}{+\sigma }_{\theta })\sin\varphi +2c\cos\varphi$$

where *c* is the soil cohesion and *φ* is the soil friction angle.

**Figure 7:**
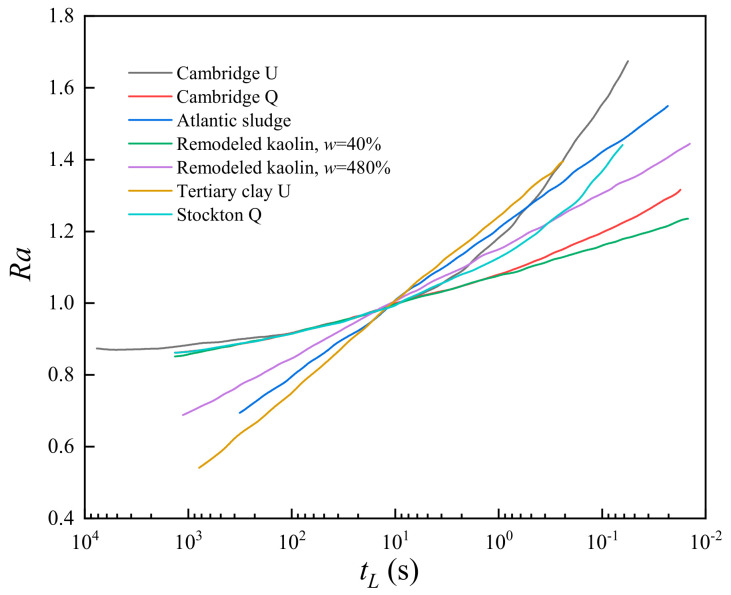
The ratio of dynamic strength to static strength versus loading time.

Due to the short duration time of the lightning strike, the wave propagation in the solid medium resulting from the lightning strike can be treated as a point explosion in an elastic half-space (Chen et al., 2017) [34]. There are some experimental data that indicate the relationship between the loading rate effect with the soil strength, as shown in Figure 7 [45]. Explosives-induced liquefaction is deemed to result from residual pore water pressure increases in saturated soils; meanwhile, explosions can improve the soil yield strength compared to the static loads. Under the dynamic load, the deformation hysteresis effect and poor drainage conditions of soil are closely related to the soil shear strength [46,47].

Note: *Ra* is the ratio of dynamic strength to static strength of soil, *t_L_* is the loading time, and *w* is the water content.

Explosives can induce a change in the nature of the soil. In this paper, the coefficient *λ* is induced to improve the soil yield strength. Substituting the coefficient *λ* into Equation (9):

(28) $$\lambda^{-1}{(\sigma }_{r}{-\sigma }_{\theta }{)=(\sigma }_{r}{+\sigma }_{\theta })\sin\varphi +2c\cos\varphi \;$$

The peak stress during the propagation of a stress wave can be given by Pan et al. [48]:

(29) $$\sigma_{r}=\frac{\sigma_{m}}{{\;r\;¯}^{a}}$$

where *σ_m_* is the initial peak pressure in the hole wall, $\;r\;¯=\frac{r}{r_{0}}$, *r*_0_ is the hole radius, and *a* is the attenuation coefficient.

Substituting Equations (26) and (27) into Equation (29):

(30) $${\left(\frac{r_{2}}{r}\right)}^{2}(\sigma_{r2}-\sigma_{re})=\lambda (\sigma_{re}+c\cos\varphi )$$

According to the continuity of the stress boundary conditions, *r* = *r*_2_, the radial stress at the outer boundary of the plastic zone is equal to the radial stress at the inner boundary of the elastic zone. The radius of the plastic zone can be acquired by substituting *r* = *r*_2_ and Equation (30) into Equation (31) as

(31) $$r_{2}={\left[\frac{\sigma_{m}r_{0}^{a}}{(\lambda \sin\varphi +1{)\sigma }_{re}+\lambda c\cos\varphi }\right]}^{\frac{1}{a}}$$

The soil plastic deformation zone by lightning strike can be acquired by substituting *P* into Equation (32) as

(32) $$r_{2}={\left[\frac{{Pr}_{0}^{a}}{(\lambda \sin\varphi +1{)\sigma }_{re}+\lambda \cos\varphi }\right]}^{\frac{1}{a}}$$

The two-stage approach is employed to solve the radius of the soil plastic zone caused by lightning strikes. The first step, the Runge–Kutta method, is an efficient iterative method for solving nonlinear ordinary differential equations, and it is applied to solve the governing equation for shock wave pressure. The second step, in the process for calculating the radius of the soil plastic zone, has no complex numerical calculation method and it does not require iterative calculations. When the parameters are provided, the shockwave overpressure and the radius of the soil plastic zone are quickly obtained from the procedures.

**Figure 8:**
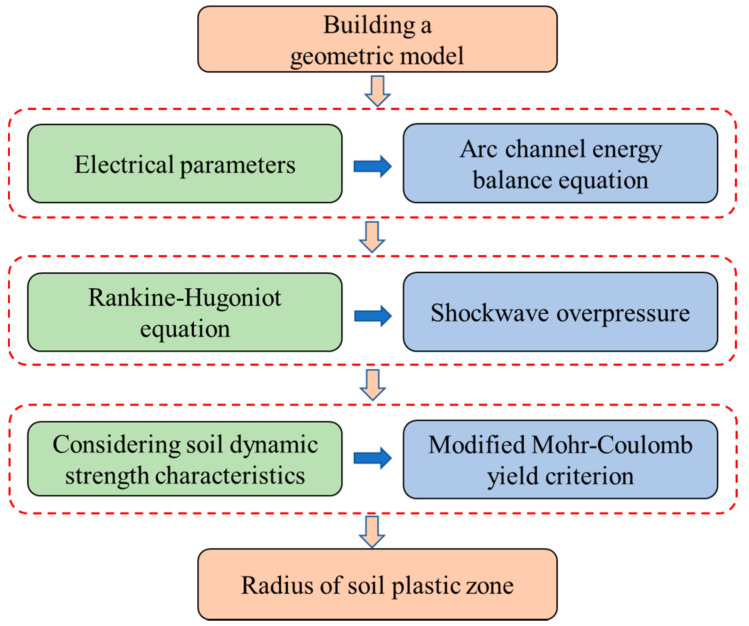
The flowchart of mathematical model and analytical solution.

However, lightning striking soil is an interdisciplinary problem about geotechnical and electrical engineering, as thus far, it has many unresolved issues, and the subject of lightning-induced soil mechanics is in the primary stage of exploration. In this paper, only the shock wave effect caused by lightning strikes is considered, which does not include the thermal effect induced by lightning strikes. When faced with real case problems, these methods can give partial results, i.e., problems caused by shock waves, but cannot handle problems caused by heat. The flowchart for solving the governing equations is as shown in Figure 8:

## 3. Comparison and Verification

As far as the authors know, neither experimental data nor an analytical model exists at present for the soil plastic zone induced by lightning strikes. Fortunately, Park et al. [30] provided some experimental data about shock wave pressure induced by a pulse discharge. Thus, the model is compared with the experimental data about shock wave pressure. The parametric analysis of the soil plastic zone induced by lightning is carried out in the next section.

### Comparisons with Existing Experiment

**Figure 9:**
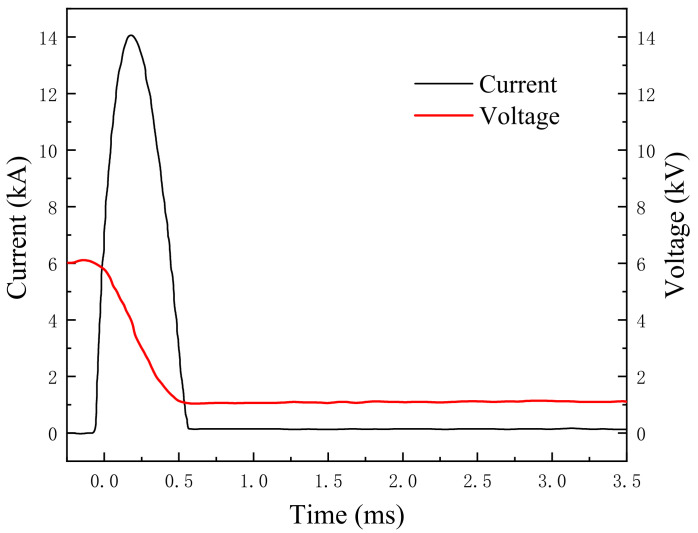
Current and voltage with time.

**Figure 10:**
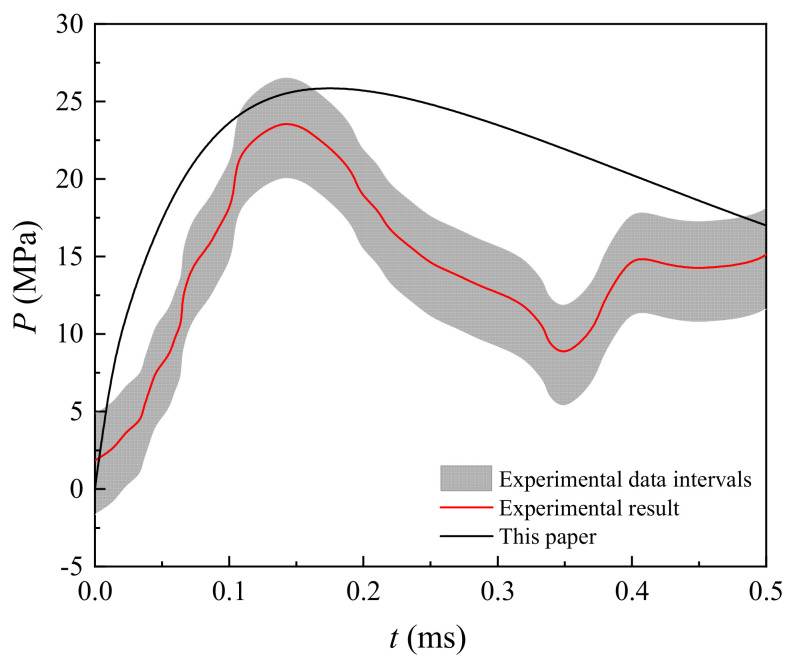
Comparison of the present analytical model and the experimental data from Park et al. (2011).

The ground borehole expansion induced by pulse discharge technology was tested by Park et al. [30]. The corresponding parameters for calculation are given in Table 1 and Figure 9. According to the data of pulse current reported by Park et al. [30], they could obtain the current as a function of time for the period 0–0.5 ms. The shock wave pressure with time calculated by the proposed analytical model has some inaccuracy with the existing experiment results, while the peak pressure and the trends are in good agreement with the existing experimental results (see Figure 10), indicating that the present model can perform well in depicting shock wave pressure induced by a pulse discharge.

## 4. Parameter Analysis

The soil parameters are provided by Xiao et al. [49], as shown in Table 2.

### 4.1. Properties of Plasma Channel Expansion

**Figure 11:**
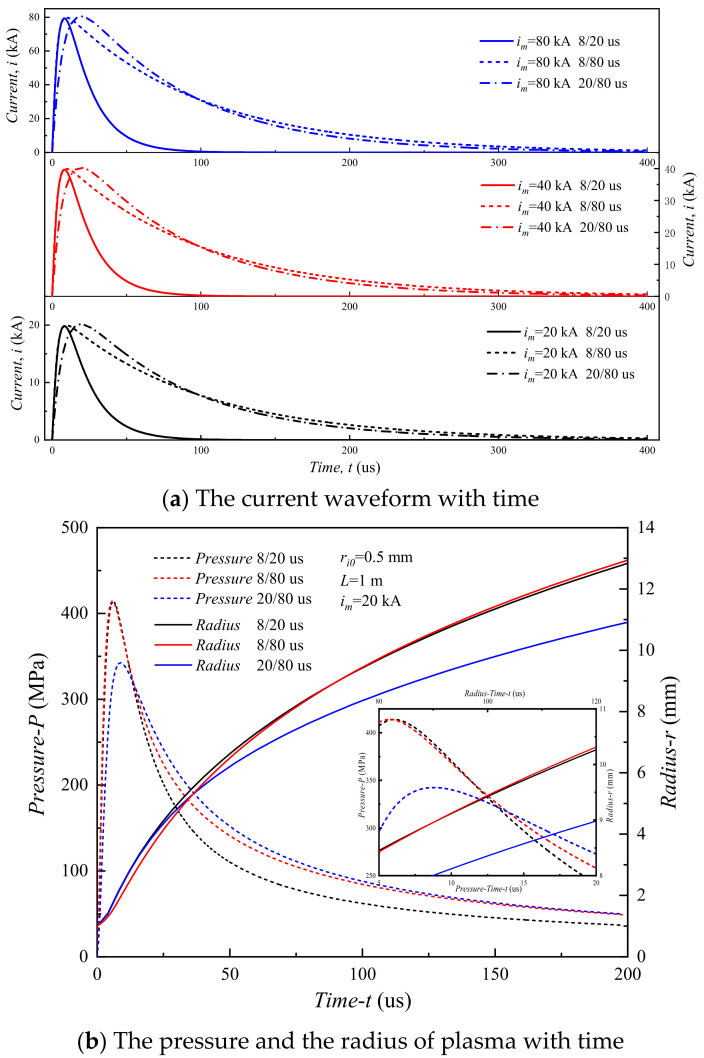
Properties of plasma channel expansion.

Figure 11a presents the current waveform with time from 0 to 400 µs for three typical systems. Comparing the waveform at 8/20 µs with that at 8/80 µs, that at 8/80 µs has a slower decline. As shown in Figure 11b, the pressure increases followed by a decrease with time, and the radius of the plasma increases monotonically with time. For the waveform of 8/20 µs, the peak pressure is 414.3 MPa, the corresponding peak pressure is 414.2 MPa for the waveform of 8/80 µs, and the peak pressure is 342.6 MPa for the waveform of 20/80 µs. In other words, the wavefront time is a major factor to peak pressure, while the half-value time is a minor factor to peak pressure.

### 4.2. Influence of the Initial Radius of Plasma Channel

**Figure 12:**
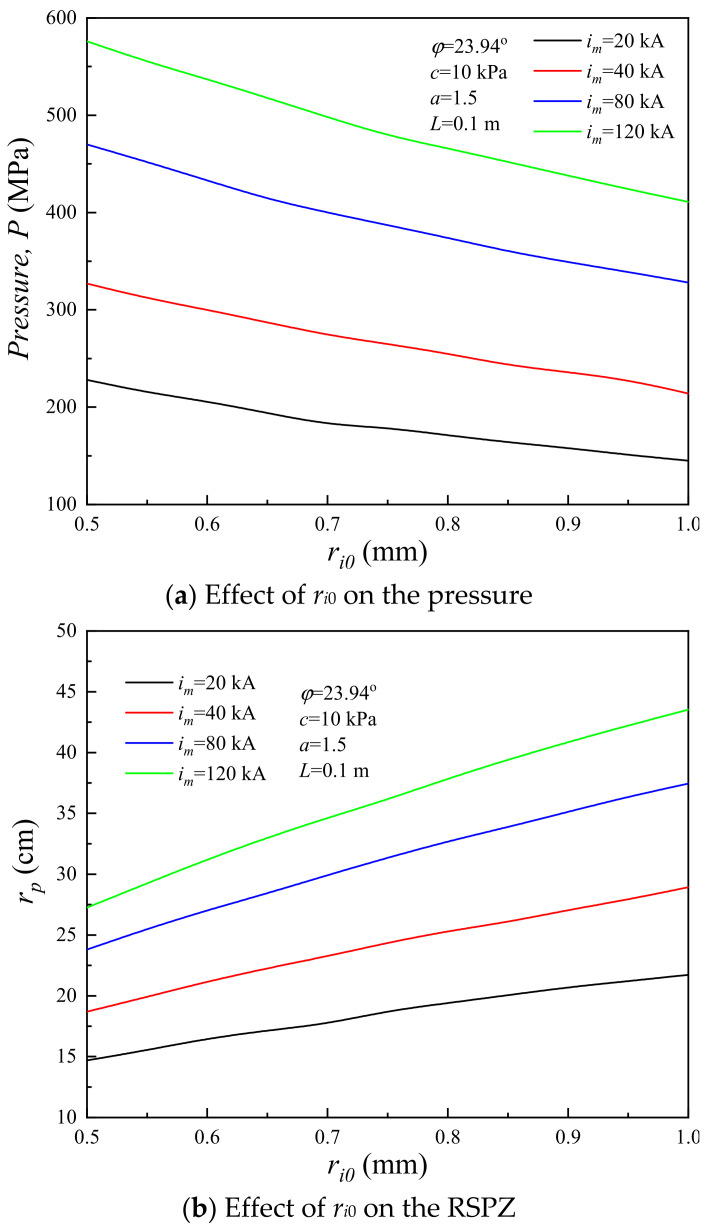
Influence of the initial radius of plasma channel.

Lightning as a source of dynamic load acts on the soil, wherein the initial radius of plasma is an important component for channel expansion [50]. To study the influence of the initial radius of plasma on the soil plastic zone induced by lightning strikes, the RSPZ of the corresponding system is calculated with *r_i_*_0_ from 0.5 mm to 1.0 mm [50]. Figure 12a presents the peak of shockwave pressure curves with *r_i_*_0_ increasing from 0.5 mm to 1.0 mm for four typical systems. The theoretical peak pressure induced by lightning decreases monotonically with the initial radius of plasma. Therefore, Figure 12b presents the RSPZ curves increasing monotonically with *r_i_*_0_. For *r_i_*_0_ = 0.5 mm, the RSPZs of *i_m_* = 20 kA, 40 kA, 80 kA, and 120 kA are 14.69 mm, 18.69 mm, 23.80 mm, and 27.25 mm, respectively, and the corresponding RSPZs are 21.73 mm, 28.93 mm, 37.45 mm, and 43.53 mm for *r_i_*_0_ = 1.0 mm. Namely, with *r_i_*_0_ increasing by 100% (from 0.5 mm to 1.0 mm), the RSPZ increases by 47.9–59.7%. In conclusion, the initial radius of plasma has a significant influence on the RSPZ induced by lightning strikes.

### 4.3. Influence of the Plasma Channel Length

**Figure 13:**
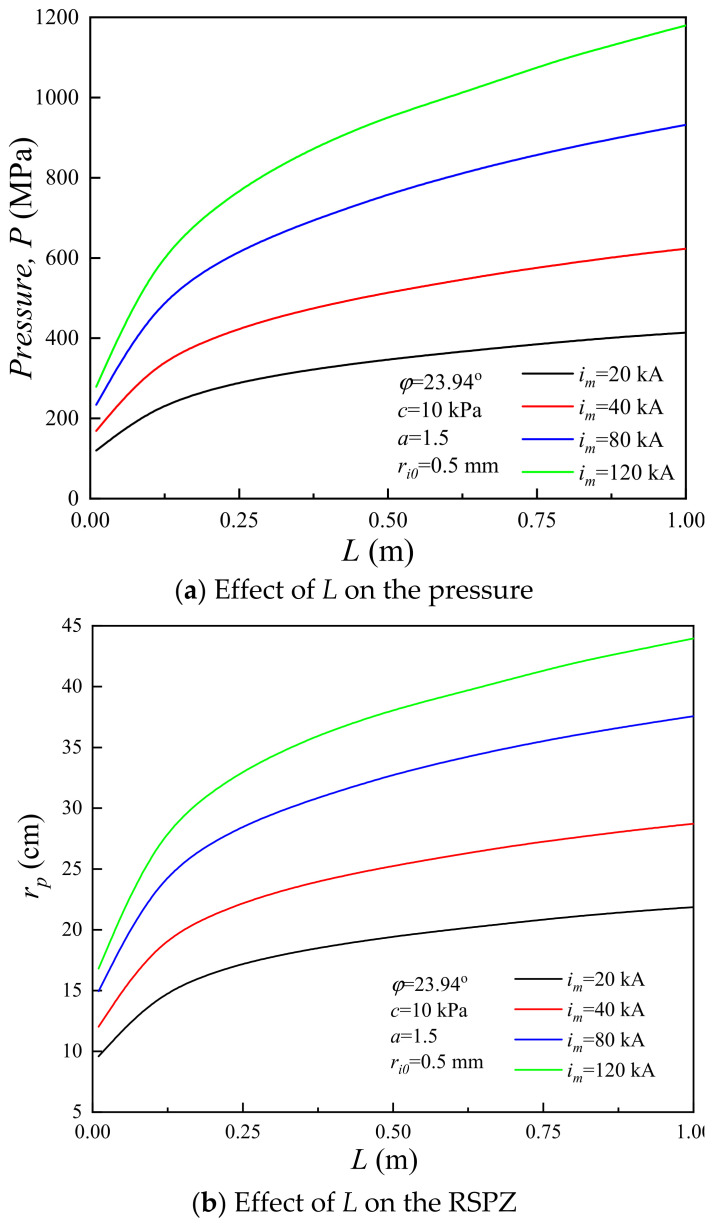
Influence of the plasma channel length.

The length of plasma is an important component for channel expansion. However, the plasma length is uncertain [16]. To study the influence of the plasma length on the soil plastic zone induced by lightning strikes, the RSPZ of the corresponding system is calculated with *L* from 0.01 to 1.0 m [50]. Figure 13a presents the peak pressure increasing monotonically with plasma length. For *L* = 0.01 m, the peak pressures of *i_m_* = 20 kA, *i_m_* = 40 kA, *i_m_* = 80 kA, and *i_m_* = 120 kA are 12 MPa, 169 MPa, 234 MPa, and 279 MPa, respectively, and the corresponding peak pressures are 273 MPa, 399 MPa, 580 MPa, and 719 MPa for *L* = 0.2 m. Namely, the slopes of *P* with *L* range from 805.3 to 2315.8. For *L* = 1.0 m, the peak pressures are 414 MPa, 623 MPa, 932 MPa, and 1180 MPa, and the corresponding slopes range from 176.3 to 576.3. Figure 13b shows the RSPZ with plasma length, *L*, from 0.01 to 1.0 m for four typical systems. The RSPZ induced by lightning increases monotonically with plasma length. For *L* = 0.01 m, the RSPZs of *i_m_* = 20 kA, 40 kA, 80 kA, and 120 kA are 9.58 mm, 12.03 mm, 14.95 mm, and 16.81 mm, respectively, and the corresponding RSPZs are 16.57 mm, 21.34 mm, 27.38 mm, and 31.60 mm for *L* = 0.2 m. Namely, the slopes of *r_p_* with *L* range from 36.8 to 77.8. The corresponding RSPZs are 21.87 mm, 28.72 mm, 37.56 mm, and 43.96 mm for *L* = 1.0 m; in other words, the slopes of *r_p_* with *L* range from 6.6 to 15.5. In conclusion, the RSPZ and peak pressure rapidly increase followed by a slight increase with plasma length.

### 4.4. Influence of the Attenuation Coefficient

**Figure 14:**
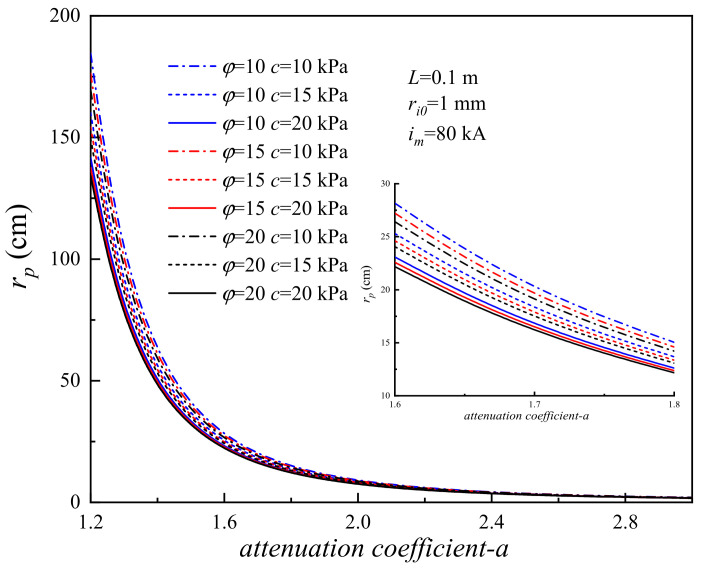
Effect of attenuation coefficient *a* on the RSPZ.

The wave propagation in solid medium resulting from the lightning strike can be treated as a point explosion in an elastic half-space (Chen et al. 2017) [34]. The filling medium is a key influencing factor that can affect the effect of decoupled charge blasting [51,52,53]. To study the influence of the attenuation coefficient on the soil plastic zone induced by lightning, the RSPZ of the corresponding system is calculated with the attenuation coefficient, *a*, from 1.2 to 3.0 [53]. Figure 14 shows the RSPZ with attenuation coefficient, *a*, from 1.2 to 3.0 for nine typical systems. The RSPZ induced by lightning decreases exponentially with attenuation coefficient.

Dividing the interval of attenuation coefficient into four segments, for attenuation coefficient *a* = 1.2, the RSPZs of (*φ* = 10°, *c* = 10 kPa), (*φ* = 10°, *c* = 15 kPa), (*φ* = 10°, *c* = 20 kPa), (*φ* = 15°, *c* = 10 kPa), (*φ* = 15°, *c* = 15 kPa), (*φ* = 15°, *c* = 20 kPa), (*φ* = 20°, *c* = 10 kPa), (*φ* = 20°, *c* = 15 kPa), and (*φ* = 20°, *c* = 20 kPa) are 184.62 mm, 160.03 mm, 141.65 mm, 176.39 mm, 154.34 mm, 137.57mm, 169.46 mm, 149.63 mm, and 134.28 mm, respectively, and the corresponding RSPZs are 28.16 mm, 25.30 mm, 23.09 mm, 27.09 mm, 24.62 mm, 22.59 mm, 26.41 mm, 24.06 mm, and 22.18 mm for attenuation coefficient *a* = 1.6. Namely, with attenuation coefficient *a* increasing by one third (from 1.2 to 1.6), the RSPZ decreases by 83.5–84.7%. For *a* from 1.6 to 2.0, increasing by 25%, the RSPZ decreases by 66.0–67.6%, and for attenuation coefficient *a* from 2.4 to 3.0, increasing by 25%, the RSPZ decreases by 51.4–52.9%. The results indicate that the attenuation coefficient would greatly improve the RSPZ.

### 4.5. Influence of the Cohesion

**Figure 15:**
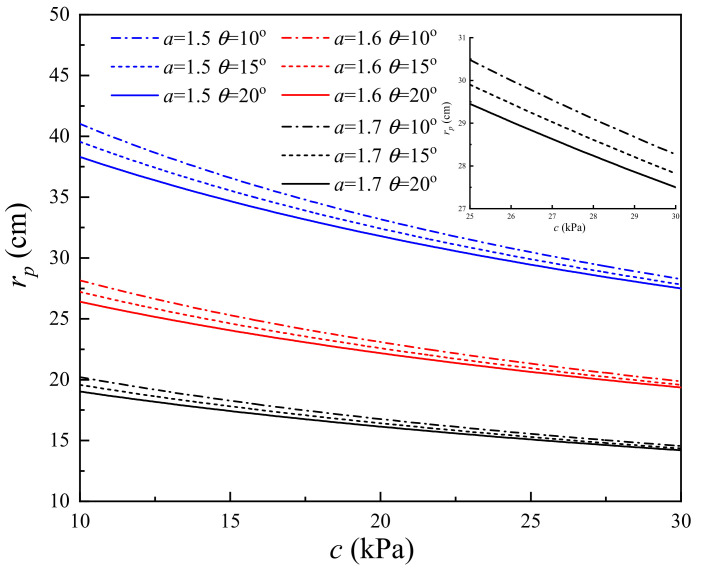
Effect of *c* on the RSPZ.

To study the influence of cohesion on the change in soil properties induced by lightning strikes, the RSPZ of the corresponding system is calculated with *c* from 10 kPa to 30 kPa. Figure 15 presents the RSPZ curves with *c* increasing from 10 kPa to 30 kPa for the nine typical systems. The theoretical RSPZ induced by lightning decreases monotonically with the soil cohesion. As shown in Figure 15, for *c* = 10 kPa, the RSPZs of (*a* = 1.5, *φ* = 10°), (*a* = 1.5, *φ* = 15°), (*a* = 1.5, *φ* = 20°), (*a* = 1.6, *φ* = 10°), (*a* = 1.6, *φ* = 15°), (*a* = 1.6, *φ* = 15°), (*a* = 1.7, *φ* = 10°), (*a* = 1.7, *φ* = 15°), and (*a* = 1.7, *φ* = 20°) are 41.02 mm, 39.55 mm, 38.31 mm, 28.16 mm, 27.22 mm, 26.41 mm, 20.21 mm, 19.57 mm, and 19.03 mm, respectively, and the corresponding RSPZs are 28.27 mm, 27.82 mm, 27.50 mm, 19.86 mm, 19.57 mm, 19.36 mm, 14.55 mm, 14.35 mm, and 14.20 mm for *c* = 30 kPa. In other words, with *c* increasing by 200% (from 10 to 30 kPa), the radius of the plastic zone decreases by 25.4–31.1%. The results reveal that the RSPZ can be improved by increasing the cohesion. (Note: *a* is the attenuation coefficient of soil and *φ* is the internal friction angle of soil.)

### 4.6. Influence of the Internal Friction Angle

**Figure 16:**
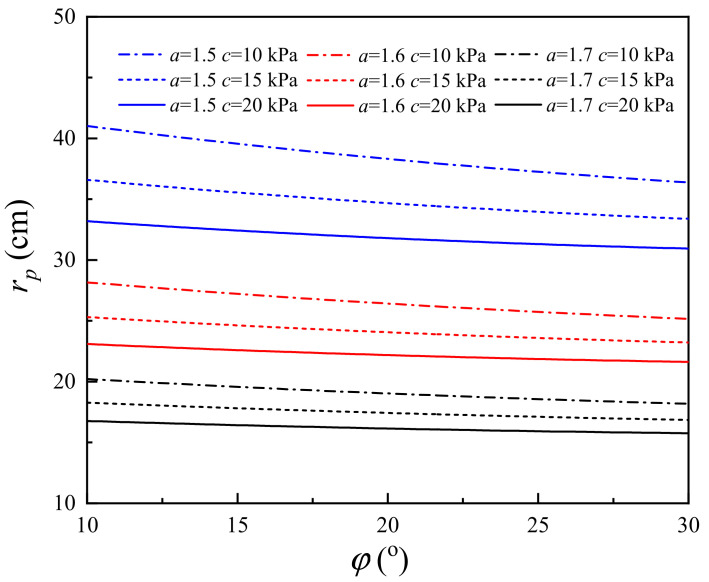
Effect of *φ* on the RSPZ.

The internal friction angle is a key parameter for soil. To study the influence of internal friction angle on the change in soil properties induced by a lightning strike, the RSPZ of the corresponding system is calculated with *φ* from 10° to 30°. Figure 16 presents the RSPZ curves with *φ* increasing from 10° to 30° for the nine typical systems. The theoretical RSPZ induced by lightning decreases monotonically with the soil cohesion. As shown in Figure 16, for *φ* = 10°, the RSPZs of (*a* = 1.5, *c* = 10 kPa), (*a* = 1.5, *c* = 15 kPa), (*a* = 1.5, *c* = 20 kPa), (*a* = 1.6, *c* = 10 kPa), (*a* = 1.6, *c* = 15 kPa), (*a* = 1.6, *c* = 20 kPa), (*a* = 1.7, *c* = 10 kPa), (*a* = 1.7, *c* = 15 kPa), and (*a* = 1.7, *c* = 20 kPa) are 41.02 mm, 36.59 mm, 33.19 mm, 28.16 mm, 25.30 mm, 23.09 mm, 20.21 mm, 18.27 mm and 16.76 mm, respectively, and the corresponding RSPZs are 36.37 mm, 33.38 mm, 30.94 mm, 25.16 mm, 23.21 mm, 21.62 mm, 18.17 mm, 16.85 mm, and 15.76 mm for *φ* = 30°. In other words, with *φ* increasing by 200% (from 10° to 30°), the radius of the plastic zone decreases by 6.3–12.8%. The results reveal that the RSPZ can be slightly improved by increasing the cohesion.

## 5. Conclusions

This paper assumes that the path of the plasma channel is a cylinder and does not have branches of channels. The assumptions in this paper are different from the actual situation. Under lightning impact, the development of the arc channel path in soil has an important impact on the improvement of lightning mitigation measures. However, the mechanism of arc channel development is not clear. With soil as a typical porous medium, the pore water in the soil will also affect the development of the arc channel. During the lightning discharge, a large amount of heat is generated. The thermal and mechanical fields are coupled, which affects the deformation of the soil and the soil properties. All the above-listed cases need to be studied one by one in the future.

This paper presents a semi-analytical solution for the peak pressure and the radius of the soil plastic zone induced by lightning strikes, based on a two-stage method. The Runge–Kutta method is applied to solve the governing equations for shockwave pressure based on mass conservation, momentum conservation, energy conservation, and the state equation of soil. The modified Mohr–Coulomb yield criterion is applied to solve the RSPZ. Therefore, if these procedures are to be implemented in real case problems by geotechnical engineers, they can also be quickly handled. The major factors dominating the shock wave pressure and the RSPZ are studied. The main conclusions are given as follows:

(1) The *r_p_* induced by lightning decreases monotonically with *c* and *φ*. Under defined conditions, *c* increases by 200% (from 10 kPa to 30 kPa), the radius of the soil plastic zone decreases from 25.4% to 31.1%, *φ* increases by 200% (from 10° to 30°), and the radius of the soil plastic zone decreases from 6.3–12.8%. With regard to *r_p_* caused by lightning strikes, *c* has a better effect on soil properties than *φ* does.
(2) Increasing the *r_i_*_0_ can reduce the *P* and increasing *r_i_*_0_ has a nonnegligible effect on *r_p_*. For example, with *r_i_*_0_ increasing by 100%, *r_p_* increases by 47.9–59.7%.
(3) The *P* and *r_p_* increase monotonically with *L*. *L* has a significant influence on *P* and *r_p_*, especially when *L* is at a relatively low level; for example, when *L* increases from 0.01 m to 0.2 m, the slopes of *r_p_* with *L* range from 36.8 to 77.8, and the slopes of *P* with *L* range from 805.3 to 2315.8.
(4) The channel radius *r_p_* significantly reduces with attenuation coefficient *a*. With attenuation coefficient *a* increasing by one third (from 1.2 to 1.6), the radius of the soil plastic zone decreases from 83.5% to 84.7%, and with attenuation coefficient *a* increasing by 25% from 2.4 to 3.0, the radius of the soil plastic zone decreases from 51.4% to 52.9%. Thus, it is necessary to improve the attenuation coefficient for decreasing soil damage induced by lightning strikes.
(5) The wavefront time is a major factor, while the half-value time is a minor factor for the shock wave pressure induced by plasma explosives. For example, when *i_m_* = 20 kA, the peak pressure of 8/80 µs is 414.2 MPa and the peak pressure of 20/80 µs is 342.6 MPa.

## Figures and Tables

**Table 1:** Material properties of cement paste.

Material	Density (kN/m^3^)	Bulk Modulus (GPa)	Young’s Modulus (GPa)	Speed of Sound (m/s)
Cement paste	24	4.87	-	1425

**Table 2:** The soil parameters.

Material	Unit Weight, *γ* (kN/m^3^)	Cohesion, *c* (kPa)	Internal Friction Angle, *φ* (°)	Elastic Modulus, *E* (MPa)	Possion’s Ratio, *υ*
Soil	18	10.5	18.9	20	0.41

## Data Availability

Not Applicable.
